# Expression differences of miR-142-5p between treatment-naïve chronic myeloid leukemia patients responding and non-responding to imatinib therapy suggest a link to oncogenic ABL2, SRI, cKIT and MCL1 signaling pathways critical for development of therapy resistance

**DOI:** 10.1186/s40164-020-00183-1

**Published:** 2020-09-26

**Authors:** Theresa Klümper, Henrike Bruckmueller, Tobias Diewock, Meike Kaehler, Sierk Haenisch, Christiane Pott, Oliver Bruhn, Ingolf Cascorbi

**Affiliations:** 1 Institute of Experimental and Clinical Pharmacology, University Hospital Schleswig-Holstein, Campus Kiel, 24105 Kiel, Germany; 2 Department of Medicine II, Haematology and Oncology, University Hospital Schleswig-Holstein, Campus Kiel, 24105 Kiel, Germany

**Keywords:** CML, Imatinib, Chemoresistance, Leukemia, miR-142-5p, miR-365a-3p

## Abstract

**Background:**

Chronic myeloid leukemia (CML) is a myeloproliferative neoplasm characterized by constitutive activity of the tyrosine kinase BCR-ABL1. Although the introduction of tyrosine kinase inhibitors (TKIs) has substantially improved patients’ prognosis, drug resistance remains one of the major challenges in CML therapy. MicroRNAs (miRNAs), a class of short non-coding RNAs acting as post-transcriptional regulators, are implicated in CML progression and drug resistance. The aim of the present study was to analyze the miRNA expression profiles of 45 treatment-naïve CML patients in chronic phase (28 peripheral blood and 17 bone marrow samples) with respect to future response to imatinib therapy.

**Methods:**

TaqMan low density arrays were used to analyze the miRNA expression pattern of the patient samples. For selected microRNAs, reporter gene assays were performed to study their ability to regulate CML associated target genes.

**Results:**

Significant lower expression levels of miR-142-5p were identified in both, peripheral blood and bone marrow samples of future non-responders suggesting a potential tumor suppressor role of this miRNA. This was supported by reporter gene assays that identified the survival, proliferation and invasion promoting CML related genes *ABL2*, *cKIT*, *MCL1* and *SRI* as targets of miR-142-5p and miR-365a-3p, the latter identified as potential biomarker in peripheral blood samples.

**Conclusion:**

MiR-142-5p and to a certain extend also miR-365a-3p were able to discriminate treatment-naïve CML patients not responding to imatinib in the course of their treatment from patients, who responded to therapy. However, further large-scale studies should clarify if the identified miRNAs have the potential as predictive biomarkers for TKI resistance.

## Background

Chronic myeloid leukemia (CML) is a clonal haemopoietic stem cell disorder characterized by a reciprocal translocation between the q-arms of chromosomes 9 and 22 thereby generating the *BCR-ABL1* fusion gene [1]. This gene is translated to the constitutively active tyrosine kinase BCR-ABL1, the determinant of CML pathophysiology [1]. Treatment of CML was revolutionized in 2001, when the first BCR-ABL1 tyrosine kinase inhibitor (TKI) imatinib was introduced [2]. Numerous studies with TKIs revealed that a deep, early molecular response leads to improved long-term outcome for patients [3, 4]. Although imatinib is extremely effective by slowing down disease progression and modulating symptoms, around 30% of patients do not respond sufficiently or develop drug resistances to standard therapy [4]. *BCR-ABL1*-dependent resistances are mainly based on amplifications or mutations in the fusion gene leading to elevated kinase activity or to attenuation of imatinib binding [1]. Although these *BCR-ABL1*-dependent mechanisms are more frequent and led to development of second and third generation TKIs, resistances can also occur independently of the fusion gene [5, 6]. Several mechanisms have been identified including activation of alternative oncogenic pathways as WNT, JAK-STAT, autophagy, and Hedgehog signaling, alterations of drug influx and efflux transporters, clonal evolution and epigenetic dysregulation, defective DNA repair mechanisms and genomic instability, inherently resistant stem cells, changes in bone marrow stromal microenvironment, and elevated levels of inhibitors of apoptosis proteins [5, 6]. Additionally, microRNAs (miRNAs), short non-coding RNAs, which post-transcriptionally modulate gene expression via mRNA degradation or translational repression, have been implicated in CML progression, response to treatment and development of TKI resistances [7, 8].

As these resistances are a major challenge in CML treatment, predictive markers are desirable to improve the selection of appropriate therapy regimes and thereby patient outcome. MiRNAs as potent regulators of gene expression are discussed as biomarkers for diagnosis, progression and drug response in various tumor types [9–12]. Previous studies analyzing miRNA expression patterns of treatment-naïve CML patients at diagnosis aiming to predict TKI resistances included only 8–12 patients and led to contradictory results. While Lavrov et al. did not find any significant differences in miRNA expression pattern between non-responding and responding patients to TKI therapy, the other three studies suggested selected miRNAs as potential predictive biomarkers for TKI response [9–12]. However, even these suggested miRNAs were inconsistent between the studies. Larger studies including more treatment-naïve CML patients are needed to clarify if selected miRNAs may have the potential to discriminate between non-responders and responders and may be considered as predictive biomarkers for TKI resistance in future.

The aim of the present study was to provide a more comprehensive miRNA expression data set of 28 peripheral blood and 17 bone marrow samples from treatment-naïve CML patients without mutations in *BCR-ABL1*. A hypothesis-free approach was selected starting with high-throughput screening of miRNA expression to identify possible new or additional miRNAs with considerable expression differences between non-responders and responders to imatinib therapy. Thereafter, the potential functional impact of the identified miRNAs was analyzed by studying their binding ability to the 3′-UTRs of critical target genes associated with CML, CML-related pathways and chemoresistance using reporter gene assays.

## Methods

### Patient samples

Peripheral blood (n = 28) or bone marrow samples (n = 17) were obtained from 45 CML patients at the time of diagnosis by the Department of Medicine II, University Hospital Schleswig–Holstein, Kiel, Germany. The patients (28 males and 17 females) had a median age of 57.9 years. (range: 19–86 years.; median age responders 57.3 years., non-responders 58.7 years). In all patients *BCR-ABL* fusion transcripts were confirmed by consensus multiplex PCR and breakpoint determination, DNA Sanger sequencing of the fusion transcript was used to verify that none of the patients did carry common mutations in the *BCR-ABL1*-gene, which are known to cause imatinib resistance [13]. According to disease progression after sampling, patients were classified into responders (n = 26) or non-responders (n = 19). Response was defined as patients reaching a major molecular response (*BCR-ABL1*/*ABL1* ratio < 1 × 10^–3^) within the first 18 months of therapy, whereas non-responders did not reach a major molecular response within this time interval [14]. Individual patients’ parameters are summarized in Table 1. The study was conducted according to the guidelines of Helsinki and was approved by the Ethics Committee of the Medical Faculty of Kiel University, Germany (reference number: D 426/03). Each patient gave his written informed consent.

**Table 1:** Patient’s characteristics including sample type, *BCR-ABL1*/*ABL1* ratio before/after imatinib therapy for responders (R) and non-responders (NR)

Patient identifier	Age (at time of sampling)	Sample type	Gender	Response	*BCR-ABL1/ABL1* ratio (at time of sampling)	*BCR-ABL1/ABL1* ratio (after 18 months)
01	62	PB	M	R	0.8	8·10^−4^
02	42	PB	M	R	0.7	4·10^−4^
03	63	PB	F	R	0.8	9·10^−4^
04	63	PB	M	R	0.8	7·10^−4^
05	45	PB	F	R	0.5	1·10^−4^
06	27	PB	M	R	0.8	7·10^−5^
07	54	PB	F	R	0.8	7·10^−4^
08	50	PB	M	R	0.4	6·10^−4^
09	47	PB	M	R	0.4	3·10^−5^
10	71	PB	M	R	0.5	2·10^−5^
11	57	PB	M	R	0.9	8·10^−4^
12	61	PB	F	R	0.7	4·10^−4^
*Median BCR-ABL1/ABL1 ratio of PB responders*					*0.75*	*5·10*^*−4*^
13	81	PB	F	NR	n.d	1·10^0^
14	79	PB	M	NR	0.8	2·10^−1^
15	71	PB	M	NR	n.d	2·10^−1^
16	46	PB	M	NR	n.d	1.1·10^0^
17	50	PB	M	NR	0.6	5·10^−1^
18	82	PB	F	NR	0.2	2·10^−1^
19	78	PB	F	NR	0.3	3·10^−1^
20	54	PB	F	NR	0.5	4·10^−1^
21	54	PB	M	NR	0.9	2·10^−2^
22	86	PB	F	NR	0.1	1·10^−1^
23	77	PB	F	NR	0.5	2·10^−1^
24	20	PB	M	NR	0.7	7·10^−1^
25	42	PB	M	NR	1.5	9·10^−1^
26	62	PB	F	NR	0.4	2·10^−1^
27	54	PB	M	NR	0.4	3·10^−2^
28	46	PB	M	NR	1.0	3·10^−1^
*Median BCR-ABL1/ABL1 ratio of PB non-responders*					*0.5*	*2.5·10*^*−1*^
29	30	BM	M	R	0.3	7·10^−4^
30	52	BM	M	R	1.1	1·10^−3^
31	71	BM	F	R	0.8	2·10^−4^
32	70	BM	F	R	0.7	7·10^−5^
33	41	BM	F	R	1.3	2·10^−3^
34	72	BM	M	R	1.0	3·10^−4^
35	56	BM	M	R	0.4	6·10^−4^
36	81	BM	M	R	1.0	1·10^−4^
37	38	BM	M	R	0.6	1·10^−4^
38	69	BM	M	R	1.1	3·10^−4^
39	49	BM	M	R	1.3	4·10^−4^
40	71	BM	M	R	2.1	5·10^−4^
41	67	BM	F	R	1.0	8·10^−5^
42	80	BM	M	R	0.7	2·10^−4^
*Median BCR-ABL1/ABL1 ratio of BM responders*					*0.7*	*3.5·10*^*−4*^
43	72	BM	F	NR	0.7	5·10^−1^
44	43	BM	F	NR	0.6	2·10^−2^
45	19	BM	M	NR	0.7	5·10^−2^
Median *BCR-ABL1/ABL1* ratio of BM non-responders					0.7	5·10^−2^

*BM* bone marrow, *PB* peripheral blood, *n.d.* not determined at this specific timepoint

### Determination of miRNA expression profiles

White blood cells from patients’ peripheral blood or bone marrow samples were purified using the QIAmp RNA Blood Mini Kit (Qiagen, Hilden, Germany) according to the manufacturers protocol and stored in RLT-buffer (Qiagen, Hilden, Germany) or trizole (ThermoFisher Scientific, Waltham, USA) at −80 °C prior to use. Total RNA was isolated from the leukocyte fraction using the mirVana miRNA Isolation Kit (ThermoFisher Scientific) according to manufacturer’s protocol. 200 ng of total RNA were reversely transcribed using Human Megaplex Primer Pools A v.2.1 and TaqMan microRNA Reverse Transcription Kit (ThermoFisher Scientific) on the GeneAmp PCR system 9700 (ThermoFisher Scientific) according to the manufacturer’s recommendations. After pre-amplification of cDNA according to manufacturer’s protocol, the pre-amplified cDNA was loaded on TaqMan Human MicroRNA Cards A v2.1 and analyzed on the ABI Prism 7900HT Sequence Detection System (ThermoFisher Scientific) with default cycling conditions. Expression data of screened miRNAs exhibiting Ct-values < 35 were included into further analysis. MiRNA expression levels were analyzed using the ^ΔΔ^Ct method with U6snRNA as endogenous control [15]. Fold changes were calculated based on the ratio of the median of non-responders compared to the median of responders for the subsets of samples derived from peripheral blood or bone marrow.

### Target prediction

An in silico target prediction was performed using different prediction tools including TargetScan 7.2 [16], microRNA.org [17], MicroCosm Targets [18] and DIANA-microT [19] to identify potential target genes of miRNAs showing p-values ≤ 0.05 in both sample origins or the lowest overall p-value comparing non-responders to responders (hsa-miR-142-5p, hsa-miR-365a-3p). Predicted targets were analyzed for their role in CML, leukemia and cancer pathways using the Gene Name Batch Viewer of the DAVID Functional Annotation Tool (DAVID Bioinformatics Resources 6.7) [20], which allows a summarized overview of function and pathway involvement for every target gene. Target genes of interest were further evaluated by literature search focusing on their involvement in CML, leukemia, CML linked pathways and chemoresistance with a minimum of 10 publications each. These genes were transferred to further functional analysis, namely: *ABL2* (*abelson murine leukemia viral oncogene homolog 2* [21–30]), *cKIT* (*KIT proto-oncogene receptor tyrosine kinase* [4, 31–39]), *MCL1* (*myeloid cell leukemia 1* [7, 40–48]), *SHC4* (*src homology 2 domain containing family, member 4* [4, 23, 49–56]) and *SRI* (*sorcin* [50, 57–65]).

### Cloning of luciferase vectors for reporter gene assays

For in vitro validation of in silico predicted interactions between miRNAs (hsa-miR-142-5p, hsa-miR-365a-3p) and selected target genes, luciferase reporter gene assays were performed. Predicted miRNA binding regions of the 3′-UTRs of selected target genes *ABL2, cKIT*, *MCL1, SHC4*, *SRI* were cloned into the pmirGLO Dual-Luciferase miRNA Target Expression Vector (Promega, Mannheim, Germany) using the In-Fusion HD Cloning Kit (Takara Bio USA, Mountain View, CA, USA) with gene-specific primers purchased from Sigma Aldrich, Missouri, USA, according to the manufacturers recommendations (for details see Additional file 1: Table S1).

### Transient transfection and luciferase reporter gene assays

The miRNA mimics (pre-miRs) used for reporter gene assay analysis were purchased from Ambion (ThermoFisher Scientific): pre-miR-142-5p (PM10979), pre-miR-365a-3p (PM11133) and pre-miR miRNA Precursor Negative Control #1 (AM17110). For reporter gene assay experiments, HepG2 cells (DMSZ, Braunschweig, Germany) were cultured as previously described [66]. Per well of a 96-well plate, a 20 µl transfection complex consisting of OptiMEM (Gibco, ThermoFisher Scientific), siPort NeoFX transfection reagent (ThermoFisher Scientific), reporter gene plasmid DNA (100 ng/well) and the selected pre-miR or pre-miR negative control were added in two concentrations (10 nM or 25 nM) according to the manufacturer protocol. Each well was covered with 80 µl of HepG2 cell suspension (100,000 cells/ml). Medium was replaced with fresh growth medium 24 h after transfection. 48 h after transfection, reporter gene activities were measured using dual-luciferase reporter assay system (Promega, Mannheim, Germany) on a Veritas microplate luminometer (Tuner Biosystems, Sunnyvale, CA, USA). To analyse the effect of the respective miRNA on the target gene vectors, first, relative reporter gene activities were obtained by normalizing the firefly to the renilla luciferase signal in each well. Next, the firefly/renilla luciferase ratio of cells co-transfected with target gene vector and pre-miR of interest was normalized to the total median of firefly/renilla ratio of cells co-transfected with target gene vector and pre-miR negative control. The same calculation was done for firefly/renilla ratios of cells co-transfected with empty vector and pre-miR of interest or pre-miR negative control. Finally, the relative luciferase activities of target genes were normalized to the relative luciferase activities of empty vectors to exclude potential indirect miRNA-mediated effects on reporter gene promoter activity [67].

### Site-directed mutagenesis

As proof of principle for selected miRNA-target gene pairs showing a significant downregulation of relative reporter gene signal compared to the empty vector, site-directed mutagenesis of the predicted miRNA binding regions was performed to confirm the exact position of the miRNA binding site in the target 3′-UTR sequence. For this, four to six bases in the binding site were mutated. Site-directed mutated plasmid vectors were obtained from GenScript (New Jersey, USA) for the following binding regions of miR-142-5p in *ABL2* 3′-UTR (MUT 1: TTTA → GTAC, mutation site: 3918–3921 bp; MUT 2: TTTA → CGCG, mutation site: 4247–4250 bp) and in *MCL1* 3′-UTR (MUT 1: TTTA → CGCG, mutation site: 2230–2233 bp) and for miR-365a-3p binding regions in *cKIT* (MUT 1: AGGG → CATA, mutation site: 1143–1146 bp; MUT 2: GGCATT → TATGCG, mutation site: 749–754 bp).

### Statistics

Differences in expression levels and in reporter gene activity were calculated using Mann–Whitney U-tests (GraphPad Software, version 7.04, San Diego California, USA). P-values < 0.05 were considered as statistically significant.

## Results

### MiRNA expression profiling of treatment-naïve CML patients revealed differences between patients non-responding or responding to imatinib therapy

MiRNA expression profiles of treatment-naïve CML patients in chronic phase not responding to imatinib in the course of their treatment were compared to the profiles of treatment-naïve patients, who responded to therapy (for details see Table 1). Molecular response was defined as *BCR-ABL1*/*ABL1* ratio < 1 × 10^–3^ within the first 18 months of therapy, whereas non-response was defined by a lack of major molecular response within this time interval. Out of a total of 374 measured miRNAs, 128 miRNAs exhibited Ct-values < 35 in the entire set of samples and were included for further analysis.

**Fig. 1:**
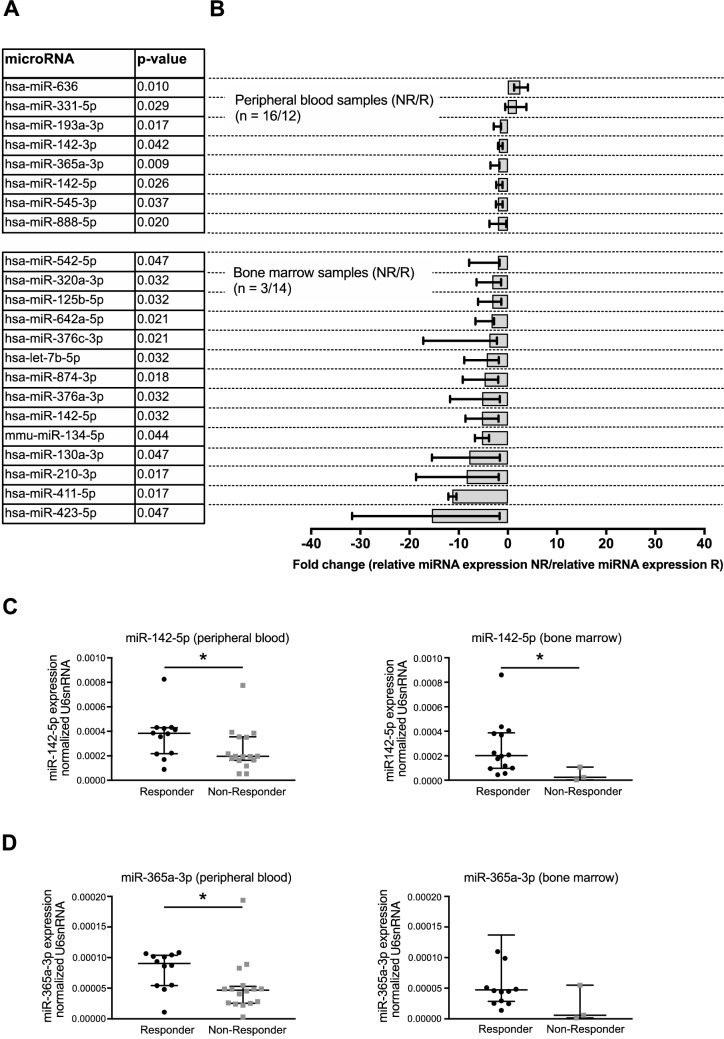
MiRNA expression profiling revealed differences between treatment-naïve CML patients non-responding vs. responding to imatinib therapy. **a** The miRNA expression profiling revealed significant differences between imatinib non-responders (NR) and responders (R) in peripheral blood and bone marrow samples. Statistical significances between NR and R were calculated using Mann–Whitney-U-tests. **b** Differences in expression levels between NR and R are presented as fold changes of relative miRNA expression levels for peripheral blood and bone marrow samples. For miR-142-5p (**c**) and miR-365a-3p (**d**) the normalized expression values are presented here, separately for the comparison of NR and R in peripheral blood and in bone marrow. MiRNA expression levels were normalized to U6snRNA using the 2^−ΔΔct^ method. Data are shown as median ± interquartile range

The analysis was performed separately for the two sample specimens, peripheral blood (PB, n = 28) and bone marrow (BM, n = 17). For the subset of samples drawn from peripheral blood, two miRNAs (miR-331-5p, miR-636) were significantly upregulated (fold change: 1.83, 2.55; p < 0.05), while six miRNAs (miR-142-3p, miR-142-5p, miR-193a-3p, miR-365a-3p, miR-545-3p, miR-888-5p) showed a significant lower expression in non-responders compared to responders (fold change range: −1.99 to −1.62; p < 0.05; Fig. 1). The analysis of bone marrow samples revealed 14 significantly downregulated miRNAs (let-7b-5p, miR-125b-5p, miR-130a-3p, miR-134-5p, miR-142-5p, miR-210-3p, miR-320a-3p, miR-376a-3p, miR-376c-3p, miR-411-5p, miR-423-5p, miR-542-5p, miR-642a-5p, miR-874-3p) in non-responders compared to responders (fold change range: −15.42 to −1.99; p < 0.05). MiR-142-5p was the only miRNA, which was significantly downregulated in both sample specimens (PB: fold change: 1.96; p = 0.02; BM: fold change: -8.63; p < 0.01) and thus appeared as most robust candidate for discrimination of non-responders from responders in our patient cohort (Fig. 1a–c). In addition, miR-365a-3p showing the most significant downregulation in peripheral blood samples of non-responders (fold change: −1.96; p < 0.01) and a tendency of lower expression levels in the non-responder group of the bone marrow sample (fold change: −7.96; p = 0.15) was chosen as additional candidate (Fig. 1a, b, d).

### Identification of potential interactions between miR-142-5p and miR-365a-3p and CML-related target genes

As the expression profiles particularly of miR-142-5p and of miR-365a-3p indicated that these two miRNAs could be candidates which are able to discriminate non-responders from responders in two different samples specimens, we aimed to investigate if the miRNAs have the potential to interact with target genes known to be involved in CML progression or drug resistance [68–70]. For this purpose, an in silico target prediction was performed using various prediction algorithms. The analysis resulted in 6004 potential target genes for miR-142-5p and in 7442 potential targets for miR-365a-3p. To focus only on the potential target genes associated with CML and drug resistance, all genes from the target prediction analysis were transferred to the Gene Name Batch Viewer of DAVID Annotation Tool and filtered for the key terms ‘CML’, ‘leukemia’, ‘chemoresistance’ and ‘pathways in cancer’. Subsequently, an extensive literature search was performed to evaluate the role of the suggested genes in context with CML. The following resulting five potential target genes with clear links to CML and CML-related pathways, cell survival and chemoresistance were selected for further interaction analysis via reporter gene assays: *cKIT*, a survival factor in CML cells, whose mutations contribute to poorer prognosis of CML patients, *MCL1*, coding for an anti-apoptotic protein often overexpressed in CML, *ABL2*, known to be upregulated in CML disease progression and potentially contributing to imatinib resistance, *SHC4*, involved in activation of BCR-ABL1 and cKIT downstream pathways, and *SRI* associated with multidrug resistance in various tumour types (for further references, see Additional file 1: Table S2) [21, 31, 40, 49, 57].

### Validation of CML-related genes as direct targets of miR-142-5p and miR-365a-3p

Reporter gene assays were performed to evaluate the potential functional impact of miR-142-5p and miR-365a-3p on the selected CML-related target genes *ABL2*, *cKIT, MCL1*, *SHC4* and *SRI* by analyzing the miRNA binding ability to target gene 3′-UTRs. The assays were conducted with two different concentrations of miR-142-5p and miR-365a-3p precursors and 3′-UTR-vector constructs of the respective target genes. To verify that decreases in reporter gene signals were originated from specific interactions between miRNA and predicted target sites, mutagenesis of target sites for selected miRNA-target gene pairs were performed including miR-142-5p—*ABL1* 3′-UTR and—*MCL1* 3′-UTR, and miR-365a-3p—*cKIT* 3′-UTR.

#### ABL2

**Fig. 2:**
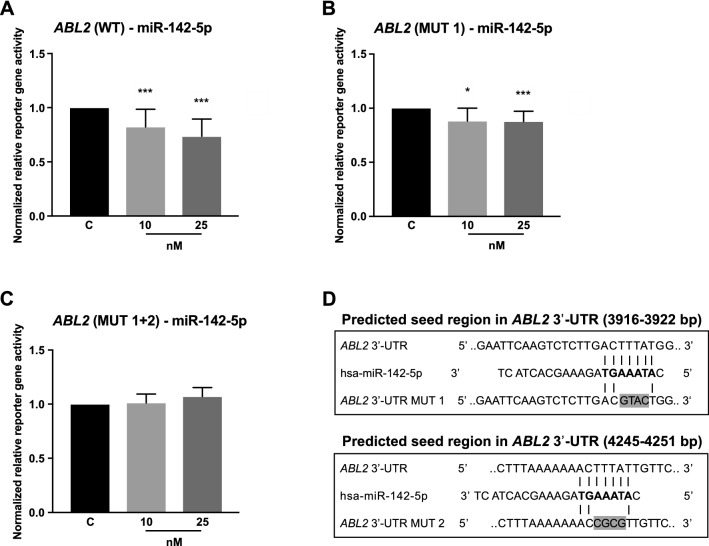
Reporter gene assays indicated a direct interaction of miR-142-5p with the *ABL2* 3′-UTR. Vectors containing the 3′-UTR of *ABL2* were co-transfected with pre-miR-142-5p in two concentrations (10 nM, 25 nM). Reporter gene activities were measured 48 h after transfection. **a** Co-transfection of vector containing the *ABL2* 3′-UTR (WT) with miR-142-5p resulted in a suppression of relative reporter gene activity by 18% [10 nM] and 26% [25 nM]. **b** Introduction of mutation (MUT 1) into the predicted binding site at positions 3918–3921 bp of *ABL2* 3′-UTR led to a minor effect. **c** Combination of MUT 1 with mutations in the predicted binding site at positions 4247–4250 bp (MUT 2) abolished the inhibitory effect of the miRNA. **d** Predicted interactions of miR-142-5p with *ABL2* 3′-UTR is indicated and mutated bases for MUT 1 and MUT 2 are highlighted in gray. All reporter gene activities (n ≥ 12 in 3 independent experiments) were normalized to activities from cells transfected with respective 3′-UTR target sequence vectors and pre-miR negative control (median ± interquartile range). Activities are shown relative to empty control vector (c) identically transfected and normalized as 3′-UTR target sequence vectors. Mann–Whitney U-test; *p ≤ 0.05, ***p ≤ 0.001

In the reporter gene assays, the 3′-UTR of the non-receptor tyrosine kinase *ABL2* was identified as target of miR-142-5p showing a significant repression of normalized reporter gene activity by 18% (10 nM, p < 0.0001) and 26% (25 nM, p < 0.0001) compared to empty vector control (Fig. 2a). As in silico tools predicted two binding sites for miR-142-5p in the *ABL2* 3′-UTR, mutations at these positions were introduced to verify the binding to these sites was responsible for the observed effect (Fig. 2b–d). While mutation at positions 3918–3921 bp (MUT 1) alone did not completely reverse the effect of the miRNA binding (Fig. 2b), the combination of MUT 1 together with mutations at positions 4247–4250 bp (MUT 2) led to a complete abolishment of the inhibitory effect of miR-142-5p (Fig. 2c).

### MCL1

**Fig. 3:**
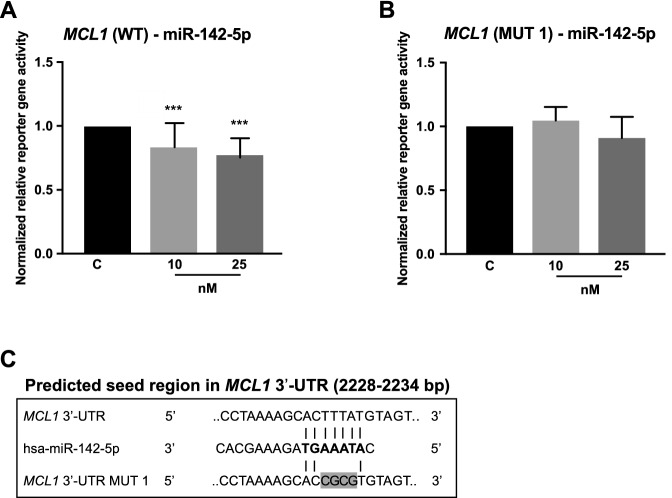
Reporter gene assays revealed a concentration-dependent interaction of miR-142-5p on *MCL1* 3′-UTR. *MCL1* 3′-UTR containing vectors were co-transfected with pre-miR-142-5p in two concentrations (10 nM, 25 nM). After 48 h, reporter gene activities were measured. **a** Normalized reporter gene activity on *MCL1* 3′-UTR (WT) was repressed by miR-142-5p 16% [10 nM] and 23% [25 nM]. **b** Mutagenesis of the predicted seed region 2230–2233 bp (MUT 1) abolished this inhibitory effect. **c** The prediction of miR-142-5p binding to *MCL1* 3′-UTR are illustrated including mutated bases highlighted in gray. All reporter gene activities (n ≥ 12 in 3 independent experiments) were normalized to activities of cells transfected with respective 3′-UTR target sequence vectors and pre-miR negative control (median ± interquartile range). Activities are shown relative to empty control vector (c) identically transfected and normalized as 3′-UTR target sequence vectors. Mann–Whitney U-test; ***p ≤ 0.001

The 3′-UTR of *MCL1* was also identified as target of miR-142-5p. The miRNA led to a significant reduction of normalized reporter gene activity by 16% (10 nM, p < 0.0001) and 23% (25 nM, p < 0.0001) compared to empty vector control (Fig. 3a). This interaction was confirmed by experiments with a mutated miR-142-5p binding region (positions 2230–2233 bp in *MCL1* 3′-UTR (MUT 1)), where the inhibitory effect of the miRNA was abolished (Fig. 3b–c).

#### cKIT

**Fig. 4:**
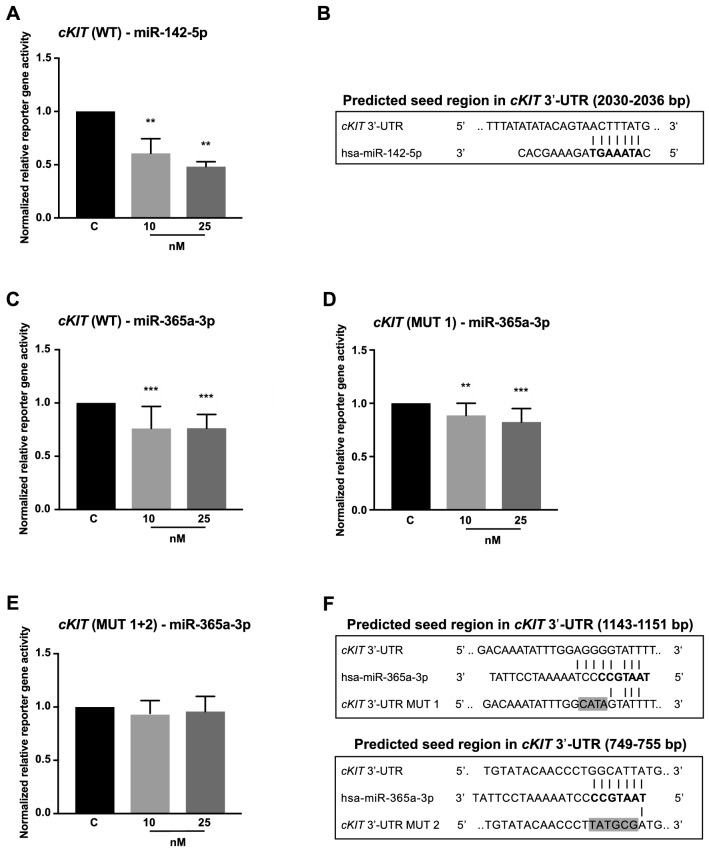
Reporter gene assay determined interactions of miR-142-5p and miR-365a-3p with *cKIT* 3′-UTR. Vectors containing *cKIT* 3′-UTR were co-transfected with pre-miR-142-5p and pre-miR-365a-3p, each in concentrations 10 nM and 25 nM. Measurement of reporter gene activities was performed 48 h after transfection. **a** Relative reporter gene activity of wild type *cKIT* 3′-UTR (WT) was repressed by miR-142-5p by 39% [10 nM] and 52% [25 nM]. **b** Predicted interactions of miR-142-5p within the binding region of *cKIT* 3′-UTR are illustrated here. **c** Co-transfection of *cKIT* 3′-UTR with miR-365a-3p resulted in repression of relative reporter gene activity by 24% [10 nM] and 24% [25 nM]. **d** The introduction of mutations in the binding region at positions 1143–1146 bp (MUT 1) slightly abolished effect on reporter gene activity. **e** Additional introduction of mutated bases at positions 749–754 bp (MUT 2) led to neutralization of the inhibitory effects of miR-365a-3p. **f** Predicted interactions of miR-365a-3p with *cKIT* 3′-UTR are depicted with mutated bases highlighted in gray. All reporter gene activities (n ≥ 12 in 3 independent experiments) were normalized to activities of cells transfected with respective 3′-UTR target sequence vectors and pre-miR negative control (median ± interquartile range). Activities are shown relative to empty control vector (c) identically transfected and normalized as 3′-UTR target sequence vectors. Mann–Whitney U-test; **p ≤ 0.01, ***p ≤ 0.001

For the receptor tyrosine kinase *cKIT,* our analysis indicated interactions with both miRNAs, miR-142-5p and miR-365a-3p, respectively. MiR-142-5p significantly repressed the normalized reporter gene activity by 39% (10 nM, p = 0.0022) and 52% (25 nM, p = 0.0022; Fig. 4a, b) and miR-365a-3p by 24% (10 nM, p < 0.0001) and 24% (25 nM, p < 0.0001; Fig. 4c–f).

As proof of principle, the predicted miRNA binding site in *cKIT* 3′-UTR were representatively analyzed for miR-365a-3p. While a mutation in the predicted binding region at positions 1143–1146 bp of *cKIT* 3′-UTR (MUT 1) had only slight effects (Fig. 4d), the combination with mutations in the binding region at 749–754 bp (MUT 2) reversed the inhibitory effect of miR-365a-3p (Fig. 4e).

#### SRI

**Fig. 5:**
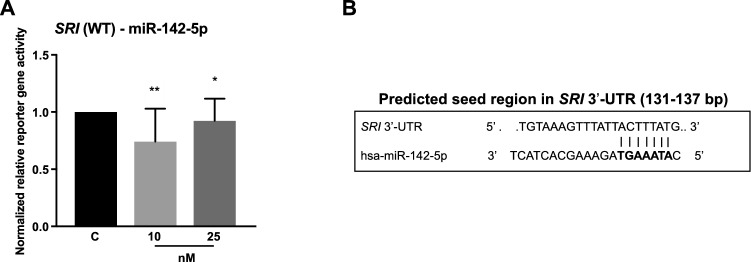
Reporter gene assays showed an interaction of miR-142-5p with *SRI* 3′-UTR. Co-transfection of the vectors containing the 3′-UTR of *SRI* with pre-miR-142-5p was performed in two concentrations (10 nM and 25 nM). 48 h after transfection, reporter gene activities were measured. **a** MiR-142-5p repressed reporter gene activity of wild type *SRI* 3′-UTR (WT) by 26% [10 nM] and 8% [25 nM]. **b** Predicted interaction of miR-142-5p with *SRI* 3′-UTR. All activities (n ≥ 12 in 3 independent experiments) were normalized to activities of cells transfected with respective 3′-UTR target sequence vectors and pre-miR negative control (median ± interquartile range). Activities are shown relative to empty control vector (c) identically transfected and normalized as 3′-UTR target sequence vectors; Mann–Whitney U-test; **p ≤ 0.01, *p ≤ 0.05

*SRI* was identified as a target of miR-142-5p, since it repressed *SRI* 3′-UTR reporter gene activity by 26% (10 nM, p = 0.0051) and 8% (25 nM, p = 0.0242; Fig. 5).

#### SHC4

Although miR-142-5p was predicted in silico to interact with the *SHC4* 3′-UTR, reporter gene experiments could not confirm any in vitro interaction (Additional file 1: Figure S1).

## Discussion

The present analysis provides miRNA expression data of 28 peripheral blood and 17 bone marrow samples from treatment-naïve CML patients at diagnosis with the aim to evaluate, if selected miRNAs could discriminate between patients being responder or non-responder to imatinib therapy, as suggested by earlier studies [9–11]. In addition, we aimed to link the identified miRNAs to oncogenic signaling pathways critical for development of therapy resistance. MiR-142-5p was significantly lower expressed in non-responders compared to responders in both sample specimens. For miR-365a-3p, a significantly lower expression was observed in PB of non-responders and a trend for lower expression in BM samples. Moreover, the miRNAs were identified to interact with the 3′-UTRs of the known CML-related oncogenes *ABL2*, *cKIT*, *MCL1* and *SRI*.

As drug resistance still remains a challenge in CML treatment, predictive biomarkers would help to optimize patients’ therapy. MiRNAs are frequently discussed as potential candidates for these biomarkers [12, 71, 72]. So far, three out of the four studies analyzing miRNA expression pattern of treatment-naïve CML patients at time of diagnosis suggested selected miRNAs as potential predictive markers for TKI resistance [9–12]. However, these studies showed varying results. Jurkovicova et al. found a total of 70 differentially expressed miRNAs between treatment-naïve non-responders and responders, José-Enériz et al*.* identified 19 miRNAs and Mosakhani et al. detected only one miRNA, miR-181c-5p, to be significantly down-regulated in non-responders compared to responders [9–11]. Furthermore, Lavrov et al*.* did not find any miRNA, whose expression pattern could discriminate between non-responds and responds to TKI therapy at time of diagnosis [12]. This inconsistency of study results might be based on factors such as differences in study design including diverse sample origin (peripheral blood or bone marrow), differences in patient cohorts, e.g. age or gender as well as the usage of various analysis platforms [73]. Another specifically important factor for the discordance in study results might be the low number of study subjects (n = 8–12), which may lead to small sample size errors characterized by increasing numbers of false positive, but also false negative results [73].

To target these shortcomings, the present study included larger numbers of samples for both major sample origins (peripheral blood and bone marrow) obtained from treatment-naïve CML patients in chronic phase classified as non-responders or responders to imatinib therapy according to molecular response during the first 18 months of treatment initiation. An unbiased, hypothesis-free approach was selected for miRNA analysis, which provided not only the possibility to confirm miRNAs described in earlier studies but enables also to identify new or additional ones. In contrast to earlier studies, we compared the miRNA expression pattern of both sample specimens with the aim to identify miRNAs whose expression pattern discriminates both patient’s groups irrespective of the sample origin, an important characteristic for a potential future biomarker. As expected, samples from both specimens exhibited differences in their miRNA expression pattern between non-responders and responders. However, by our approach we were able to identify miR-142-5p as most robustly expressed miRNA showing significant expression differences in both, peripheral blood and bone marrow samples. Although the expression differences were modest, they could substantially influence cellular processes [74]. This is also supported by the recent study of Alves et al*.* in CML patients*,* which described slight miRNA expression differences between TKI non-responders and responders at diagnosis that became more pronounced after six and twelve months of therapy [75].

While miR-142-5p is described as master regulator of hematopoiesis, its role in cancer seems to depend on the specific context [70]. In non-small cell lung and breast cancer tissue, an overexpression of miR-142-5p was associated with cell growth and progression, whereas low expression levels were observed in CLL [70, 76, 77]. In our study, miR-142-5p was downregulated in peripheral blood and bone marrow samples of non-responders, along with miR-let-7b-5p, miR-320a-3p and miR-130a-3p, all described to target CML relevant oncogenes as *BCR-ABL1* [7, 78]. Decreased expression levels of miR-365a-3p seem also to result in various effects depending on the cancer type. While miR-365a-3p inhibits carcinogenesis in epidermal squamous cell carcinomas, it has been characterized as onco-miR in laryngeal squamous cell carcinoma [68, 69].

In the second part of this study, initial functional analyses were performed to investigate the potential impact of these two miRNAs on CML signaling pathways. After identification of target genes of miR-142-5p and miR-365a-3p with link to CML pathogenesis and therapy resistance, reporter gene assays were performed. MiR-142-5p was confirmed to interact with the *ABL2*, *MCL1, cKIT* and *SRI* 3′-UTR*,* while miR-365a-3p, found as significant marker in peripheral blood samples, was identified to interact with the 3′-UTR of *cKIT*. These miRNA-target gene interactions, together with the low expression levels of miR-142-5p and miR-365a-3p in patients non-responding to imatinib therapy, suggest a tumor suppressor role in CML context.

**Fig. 6:**
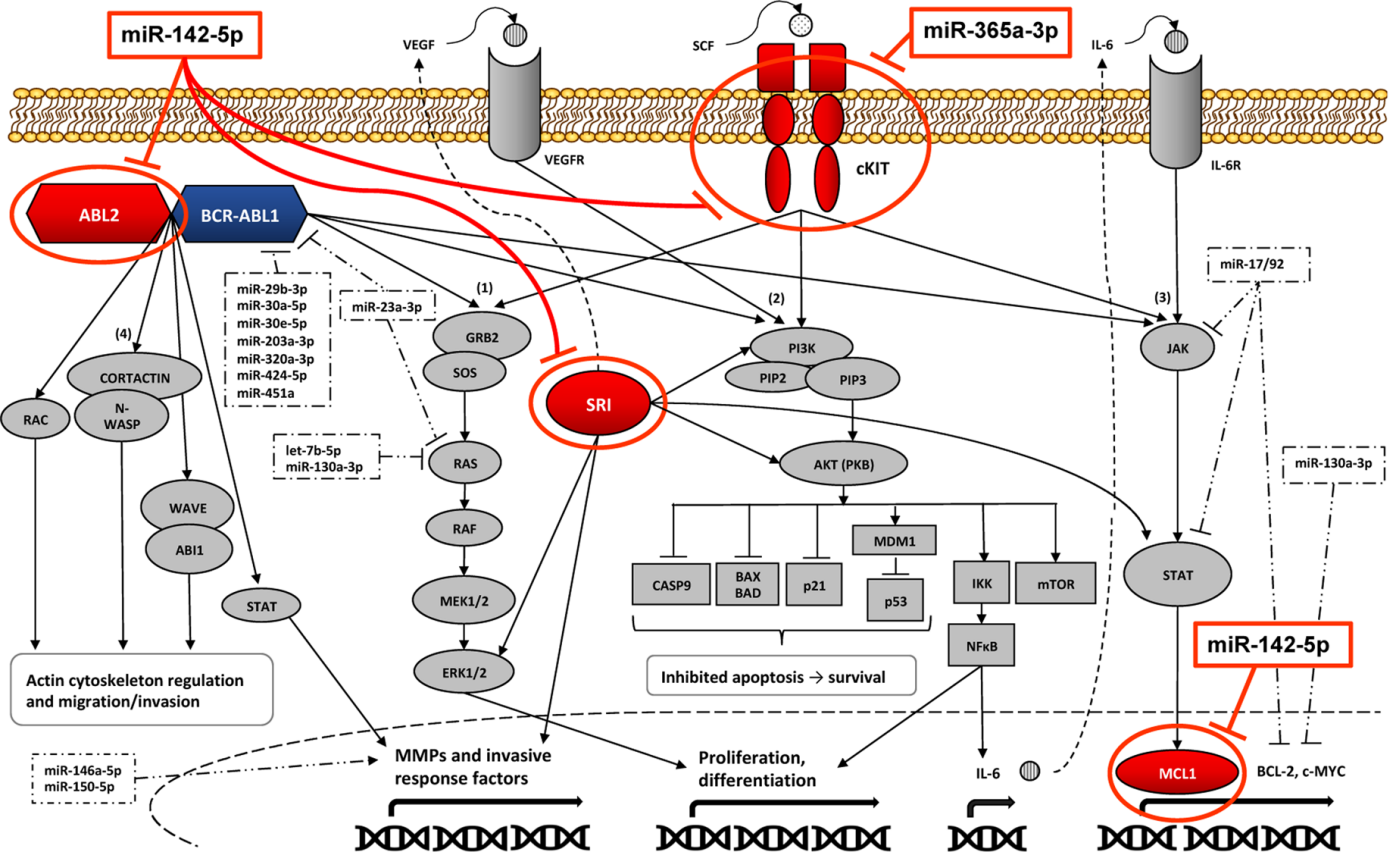
Hypothesized role of miR-142-5p and miR-365a-3p in CML signaling. Schematic representation of the hypothesized effect of miR-142-5p and miR-365a-3p (indicated by red boxes) on CML linked pathways by targeting *ABL2*, *cKIT*, *MCL1* and *SRI* (indicated by red circles). Ligands as EGFR, FGFR, PDGFR and SCF bind to cKIT and activate downstream signaling cascades, including RAS/RAF/MEK/ERK, PI3K/AKT and JAK2 [32–34]. In the RAS/RAF/MEK/ERK pathway [1], the GRB2/SOS complex activates a phosphorylation cascade that regulate proliferation and apoptosis[52]. PI3K/AKT pathway **(2)** is activated e.g. by growth factors and stimulates PI3K, mediating the conversion of PIP2 to PIP3. PIP3 translocates AKT to plasma membrane, where AKT regulates numerous proteins associated with proliferation, metabolism, apoptosis, including NFκB inducing inflammatory cytokines like IL-6[51, 88]. IL-6 activates the JAK/STAT pathway **(3)** where STAT translocates to nucleus, stimulating transcription of genes linked to cell proliferation, differentiation, survival and immune function as VEGF and BCL-2*-*family members like MCL1[43]. Upregulation of cKIT due to decreased miR-142-5p and miR-365a-3p expression might result in augmented proliferation, differentiation and survival. Downregulation of miR-142-5p might lead to upregulation of MCL1, facilitating cell survival. Both ABL1 and ABL2 stimulate RAC, Cortactin/N-WASP and WAVE/ABI1 **(4)** promoting migration and invasion through lamellipodia protrusion and actin/myosin filament contraction[24]. Downregulation of miR-142-5p would support metastasis through upregulation of ABL2. Sorcin regulates JAK/STAT, RAS/RAF/MEK/ERK, PI3K/AKT pathways and MMP expression, hence downregulation of miR-142-5p might increase sorcin and promote proliferation, differentiation, cell motility and survival[50, 62, 63]. Selection of miRNAs known to be linked to CML are illustrated in dashed boxes. MiR-29b-3p, miR-30a-5p, miR-30e-5p, miR-203-3p, miR-320a-3p, miR-424-5p, miR-451a and miR-23a-3p target *BCR/ABL1*, while miR-23a-3p, let-7b-5p and miR-130a-3p inhibit *RAS *[6, 7, 78]. MiR-130a-3p furthermore regulates *BCL-2* and *MCL1* expression [7]. Besides inhibiting *BCL-2*, miR-17/92 targets *JAK2*/*STAT5 *[7]. MiR-146a-5p and miR-150-5p contribute to CML carcinogenesis by upregulating MMPs [89]. Full gene names listed in Additional file 1: Table S3

We integrated our findings into the complex CML pathway network and tried to propose potential mechanisms involving miR-142-5p and miR-365a-3p (Fig. 6). *ABL2*, a member of the Abelson family of non-receptor tyrosine kinases, was identified as target of miR-142-5p in the present study and is known to be upregulated in CML disease progression [21–23]. By regulating cytoskeleton rearrangement, invasiveness of neoplastic cells and expression of matrix metalloproteinases (MMPs), it plays a substantial role in the BCR-ABL1 signaling network and is described to be involved in the acquisition of chemotherapy resistance in diverse solid tumors [23, 24].

MiR-142-5p was also identified to interact with *MCL1*, a member of the anti-apoptotic *Bcl-2* family, which is involved in the JAK/STAT, RAS/RAF/MEK/ERK and STAT5 signaling pathways [41]. *MCL1* is a *BCR-ABL1*-dependent survival factor, constitutively expressed in primary CML cells, that has been implicated in CML pathogenesis, whereas downregulation of *MCL1* by antisense oligonucleotides is associated with increased sensitivity of K562 cells to imatinib [40]. In various cancer contexts, *MCL1-*overexpression was described as possible key factor for therapy resistances [41, 42].

*SRI* is also linked to multidrug resistances in a variety of tumor types and was here also identified as target of miR-142-5p [58–60]. An overexpression of the gene product sorcin was correlated to poor clinical outcome and suggested to contribute to drug resistance of AML patients [61]. Sorcin contributes to tumor growth and metastasis via regulation of the JAK/STAT, RAS/RAF/MEK/ERK and PI3K/AKT pathways, as well as via regulation of MMP expression [50, 62, 63, 79].

Moreover, *cKIT* was identified in the present study as a target of both significantly downregulated miRNAs, miR-142-5p and miR-365a-3p. Beside BCR-ABL1 inhibition, imatinib provides inhibitory effects on cKIT signaling, which is discussed to be important to achieve the maximal effect of the drug [4, 80]. cKIT induces the PI3K/AKT pathway, which leads to NF-κB activation, antiapoptotic and pro-proliferative effects [32]. cKIT is also known to induce the RAS/RAF/MEK/ERK and JAK2 pathways and thereby regulates proliferation and cell death [33, 34].

Only for the miRNA–gene pair miR-142-5p–*SHC4*, results of reporter gene assays did not indicate any interaction. Such a finding is not unexpected, as even target prediction algorithms may have a false positive rate of 20–50%. This result rather highlights the need of experimental validation of in silico predicted miRNA–gene interactions [81].

Taken together, potential mechanisms, how decreased expression of miR-142-5p and miR-365a-3p might contribute to development of imatinib resistance in CML patients could be suggested based on our findings and on literature data. However, it must be taken into account that these interaction data were generated in an in vitro setup. Therefore, further functional studies are needed, including the quantification of mRNA and protein levels of target genes and its affected pathways in patients. Due to lack of patient material, it was not possible to perform these experiments in the present study.

Before truly considering these miRNAs as candidates for predictive biomarkers, additional aspects need to be considered. Although we were able to include more patients than previous studies, the overall sample size is still small, and a certain degree of variability in miRNA expression levels was observed. Reasons for this variability might be the general biological diversity of patients, differences in miRNA expression pattern due to diverse cells compositions in sample specimens and the intratumoral heterogeneity in CML [82, 83]. As it is shown that expression levels of selected miRNAs change across different CML phases, one may assume that even within the chronic phase dynamic changes on miRNA expression pattern might occur [83]. These aspects need to be targeted in further studies. It is crucial to fully understand the role of miRNAs in TKI resistance before further consideration as potential candidates for predictive biomarkers. This is of particular importance, because miRNAs lack an exclusive link to one target gene or disease and thus lack the high specificity, which is a requested feature of biomarkers [72, 84]. Future studies should also include further investigations on the role of long non-coding RNAs in imatinib resistance as studies indicate that imatinib resistance can be modulated by long-non coding RNAs which target miRNAs thereby changing the expression of target associated with drug resistance like ABCB1 or ABCC2 [85–87].

## Conclusion

In summary, this comprehensive analysis provides first evidence for a potential tumor suppressor role of miR-142-5p and less pronounced of miR-365a-3p in CML progression and TKI drug response. Furthermore, it highlights also the need of further large-scale prospective, randomized trials to replicate findings of miRNA expression analysis and thereby identify the most robust miRNA candidates which might have a potential for future predictive biomarkers for TKI resistance.

## Supplementary information

**Additional file 1: Table S1.** Vector constructs used in reporter gene assays for target gene evaluation of the selected miRNAs.** Table S2.** Selected target genes of miR-142-5p and miR-365a-3p, which were analyzed in reporter gene-assays including a summary of their relevance in CML.** Table S3.** Full terms of abbreviations.** Figure S1.** Reporter gene assays indicated no interaction between miR-142-5p and SHC4 3’-UTR. The SHC4 3’-UTR containing vector was co-transfection with pre-miR-142-5p and reporter gene activity were measured after 48 h. (A) The reporter gene assay resulted in no significant suppression of relative reporter gene activity. (B) Predicted interaction between miR-142-5p and SHC4 3’-UTR is shown here. All activities (n ≥ 12, median ± interquartile range) were normalized to activities of cells transfected with respective 3′-UTR target sequence vectors and pre-miR negative control. Activities are shown relative to empty control vector (c) identically transfected and normalized as 3′-UTR target sequence vectors, Mann-Whitney U-test.

## Data Availability

Data available on request.
