# Supplementary material for: Expression differences of miR-142-5p between treatment-naïve chronic myeloid leukemia patients responding and non-responding to imatinib therapy suggest a link to oncogenic ABL2, SRI, cKIT and MCL1 signaling pathways critical for development of therapy resistance
Source: Exp Hematol Oncol. 2020 Sep 26;9:26. doi: 10.1186/s40164-020-00183-1 (PMC7519530; doi:10.1186/s40164-020-00183-1)
Supplement: Supplementary file 1 — Additional file 1: Table S1. Vector constructs used in reporter gene assays for target gene evaluation of the selected miRNAs. Table S2. Selected target genes of miR-142-5p and miR-365a-3p, which were analyzed in reporter gene-assays including a summary of their relevance in CML. Table S3. Full terms of abbreviations. Figure S1. Reporter gene assays indicated no interaction between miR-142-5p and SHC4 3’-UTR. The SHC4 3’-UTR containing vector was co-transfection with pre-miR-142-5p and reporter gene activity were measured after 48 h. (A) The reporter gene assay resulted in no significant suppression of relative reporter gene activity. (B) Predicted interaction between miR-142-5p and SHC4 3’-UTR is shown here. All activities (n ≥ 12, median ± interquartile range) were normalized to activities of cells transfected with respective 3′-UTR target sequence vectors and pre-miR negative control. Activities are shown relative to empty control vector (c) identically transfected and normalized as 3′-UTR target sequence vectors, Mann-Whitney U-test. [file 40164_2020_183_MOESM1_ESM.docx]

**Supplement to**

**Expression differences of miR-142-5p between treatment-naïve chronic myeloid leukemia patients responding and non-responding to imatinib therapy suggest a link to oncogenic ABL2, SRI, cKIT and MCL1 signaling pathways critical for development of therapy resistance**

Theresa Klümper ^§1^, Henrike Bruckmueller ^§1^, Tobias Diewock ^1^, Meike Kaehler ^1^, Sierk Haenisch ^1^, Christiane Pott ^2^, Oliver Bruhn *^1^, Ingolf Cascorbi *^1^

**Table S1.** Vector constructs used in reporter gene assays for target gene evaluation of the selected miRNAs.

| **Target mRNA** | **Gene Accession** | **Name of vector construct** | **Length 3’-UTR [bp]** | **Length of 3’-UTR-insert [bp]** | **Position of vector insert in the 3’-UTR**  **[bp]** | **Sequence F-Primer (F) 5‘-3‘**  **Sequence R-Primer (R) 5‘-3‘** | **Annealing temperature** | **Elongation time** | **microRNA predicted to bind to 3’-UTR** | **Position of predicted**  **binding sites and the mutated sites (MUT) in 3’-UTR** |
| --- | --- | --- | --- | --- | --- | --- | --- | --- | --- | --- |
| *ABL2* | NM_007314.3 | *ABL2*-3‘-UTR | 8391 | 659 | 3746-4404 | **F:**GCTCGCTAGCCTCGACCAGTAATCTCGCCAGGTGT  **R:**CGACTCTAGACTCGAATTTTCAGGTACCCCACAGGG | 68 °C | 100 s/cycle 3200 sec | hsa-miR-142-5p | 3916-3922^A,B,D^ (MUT 1: 3918-3921)  4245-4251^A,B,D^ (MUT 2: 4247-4250) |
| *cKIT* | NM_000222.2 | *cKIT*-3‘-UTR | 2158 | 1826 | 260-2085 | **F:**GCTCGCTAGCCTCGAAGAGGGAGGTATGGACTGGG  **R:**CGACTCTAGACTCGAACGTGGAACACCAACATCCTT | 72 °C | 100 s/cycle 3200 sec | hsa-miR-142-5p | 2030-2036^A,B,D^ |
|  |  |  |  |  |  |  |  |  | hsa-miR-365a-3p | 1143-1151^C^ (MUT 1: 1143-1146)  749-755^A,B,C^ (MUT 2: 749-754) |
| *MCL1* | NM_021960.4 | *MCL1*-3‘-UTR | 2824 | 1891 | 454-2344 | **F:**GCTCGCTAGCCTCGAAGCCTCAGTACTGTACAAGGGA  **R:**CGACTCTAGACTCGAAAGTGGGGCCCCTAAAAACC | 72 °C | 100 s/cycle 3200 sec | hsa-miR-142-5p | 2228-2234^A,B,D^ (MUT 1: 2230-2233) |
| *SHC4* | NM_203349.3 | *SHC4*-3‘-UTR | 2234 | 1863 | 316-2178 | **F:**GCTCGCTAGCCTCGACTTGAATGAACTGCTGGGGC  **R:**CGACTCTAGACTCGAGGCAAATGGATTTCCACTTTAAGAC | 68 °C | 100 s/cycle 3200 sec | hsa-miR-142-5p | 492-498^A,B^ |
| *SRI* | NM_003130.3 | *SR*I-3‘-UTR | 1361 | 1269 | 42-1310 | **F:**GCTCGCTAGCCTCGAGCTCTCCTTTGCTTGTCCTCT  **R:**CGACTCTAGACTCGATGGTAAGTTTCACACTGCATCC | 72 °C | 100 s/cycle 3200 sec | hsa-miR-142-5p | 131-137^A,B,C,D^ |

^A^TargetScan 7.2; ^B^microRNA.org; ^C^MicroCosm Targets; ^D^DIANA-microT

**Table S2.** Selected target genes of miR-142-5p and miR-365a-3p, which were analyzed in reporter gene-assays including a summary of their relevance in CML.

| Gene symbol | Gene name | Function/relevance | References |
| --- | --- | --- | --- |
| *ABL2* | *abelson murine leukemia viral oncogene homolog 2* | - fusion gene in rare leukemia (CML, T-ALL, AML) - potentially leads to lethal mastocytosis - upregulated in CML disease progression - inhibited by TKIs including imatinib, nilotinib, bosutinib, dasatinib and ponatinib - promotes migration, invasion, adhesion, phagocytosis, morphogenesis, cell growth, proliferation, survival and invasiveness via RAC, Cortactin/N-WASP, WAVE/ABI1 and MMPs in several cancers, including CML, non-small-lung cancer, prostate cancer, cervical carcinomas and breast cancer | 21–30 |
| *cKIT* | *KIT proto-oncogene receptor tyrosine kinase*;  *c-KIT* | - oncogene executing its anti-apoptotic role i. e. by inducing RAS/RAF/MEK/ERK, PI3K/AKT/mTOR and JAK2 pathways - survival factor in CML cells, mutations contribute to poorer prognosis for CML patients - mutations driven leukemia, systemic mastocytosis, gastrointestinal stromal tumor (GIST), subsets of AML and melanomas - target of imatinib, participates to imatinib response and resistance - constitutively activated CD117/autocrine SCF secretion are discussed to account for resistance to nilotinib | 4,31–39 |
| *MCL1* | *myeloid cell leukemia 1* | - anti-apoptotic function in normal and malignant tissues, overexpressed in variety of hematopoietic, lymphoid and solid cancers including CML, constitutively expressed BCR-ABL1 dependent target in CML - activated by IL-6/STAT and IL-3/PI3K pathways, regulates CML cell differentiation via STAT regulation - downregulation of *MCL1*, e. g. by MCL1 inhibitor S63845, imatinib or antisense oligonucleotides promotes apoptosis and counteracts growth and viability in (CML) cancer cells - higher cytotoxic effect of sorafenib compared to dasatinib correlates with stronger downregulation of *MCL1* in AML - linked to B-CLL cell survival and chemotherapy resistance | 7,40–48 |
| *SHC4* | *SHC* (*src homology 2 domain containing* *family*, *member 4*) Protein 4 | - Shc is phosphorylated by Src kinases, which are a target of dasatinib, bosutinib and ponatinib - Shc mediates BCR-ABL1 and cKIT activation of pathways including RAS and PI3K - phosphorylation of Shc is found in many leukemias - imatinib sensitive - Shc overexpression is associated with carcinogenesis and metastasis, e. g. in lung cancer and breast cancer - Shc expression is altered during terminal differentiation of hemopoietic cells, and in CML closely correlated to CD34^+^ antigen expression in CP and blast crisis | 4,23,49–56 |
| *SRI* | *sorcin* | - crucial role in multidrug resistance, e. g. to paclitaxel, cisplatin and doxorubicin, in diverse tumor cells including leukemia cells - inhibition of sorcin reverses metastasis, tumor growth and multidrug resistance in leukemic cells - causes invasion, migration, tumor growth and metastatic activity of cancer cells via regulation of STAT3, MAPK/ERK, VEGF, NFκB, MMPs and PI3K/AKT pathways in a variety of cancers including gastric cancer, hepatocellular carcinoma and colorectal cancer - sorcin and MDR1 gene expressions correlate positively in AML; co-amplification observed in leukemia can be used as indicator of clinical drug resistance and prognosis, at which sorcin overexpression is linked to poor clinical outcomes | 50,57–65 |

**Table S3.** Full terms of abbreviations

| **Abbreviation** | **Full term** |
| --- | --- |
| *ABI1* | *ABL interactor 1* |
| *ABL2* | *Abelson murine leukemia viral oncogene homolog 2* |
| *AKT* | *Protein kinase B* |
| *BAD* | *Bcl-2 antagonist of cell death* |
| *BAX* | *Bcl-2 associated X protein* |
| *BCL-2* | *B-cell lymphoma 2* |
| *BCR-ABL1* | *Breakpoint cluster region - Abelson murine leukemia viral oncogene homolog 1* |
| *BM* | *Bone marrow* |
| *CASP9* | *Caspase 9 / apoptosis-related cysteine peptidase* |
| *cKIT* | *KIT proto-oncogene receptor tyrosine kinase* |
| *CLL* | *Chronic lymphocytic leukemia* |
| *CML* | *Chronic myeloid leukemia* |
| *c-MYC* | *MYC proto-oncogene* |
| *EGFR* | *Epidermal growth factor receptor* |
| *ERK* | *Extracellular signal-regulated kinase* |
| *FGFR* | *Fibroblast growth factor receptor* |
| *GRB2* | *Growth factor receptor-bound protein 2* |
| *IKK* | *IκB kinase* |
| *IL-6* | *Interleukin-6* |
| *JAK* | *Janus kinase* |
| *MCL1* | *Myeloid cell leukemia 1* |
| *MDM1* | *Mdm1 nuclear protein* |
| *MEK* | *Mitogen-activated protein kinase* |
| *miRNA* | *microRNA* |
| *MMP* | *Matrix metalloproteinase* |
| *mTOR* | *Mechanistic target of rapamycin* |
| *MUT* | *Mutagenesis* |
| *NFκB* | *Nuclear factor kappa B* |
| *nM* | *Nanomolar* |
| *NR* | *Non responder* |
| *N-WASP* | *Neural Wiskott-Aldrich syndrome protein* |
| *P21* | *Cyclin-dependent kinase inhibitor 1* |
| *PB* | *Peripheral blood* |
| *PDGFR* | *Platelet-derived growth factor receptor* |
| *PI3K* | *Phosphoinositide 3-kinase* |
| *PIP2* | *Phosphatidylinositol (3,4)-bisphosphate* |
| *PIP3* | *Phosphatidylinositol (3,4,5)-trisphosphate* |
| *R* | *Responder* |
| *RAC* | *RAS-related c3 botulinum toxin substrate* |
| *SCF* | *Stem cell factor* |
| *SHC4* | *Src homology 2 domain containing family, member 4* |
| *SOS* | *Son of Sevenless* |
| *SRI* | *Sorcin* |
| *STAT* | *Signal transducer and activator of transcription* |
| *TKI* | *Tyrosine kinase inhibitor* |
| *UTR* | *Untranslated region* |
| *WT* | *Wild type* |

**Legend to Figure S1.**

**Reporter gene assays indicated no interaction between miR-142-5p and *SHC4* 3’-UTR.** The *SHC4* 3’-UTR containing vector was co-transfection with pre-miR-142-5p and reporter gene activity were measured after 48 h. (A) The reporter gene assay resulted in no significant suppression of relative reporter gene activity. (B) Predicted interaction between miR-142-5p and *SHC4* 3’-UTR is shown here. All activities (n ≥ 12, median ± interquartile range) were normalized to activities of cells transfected with respective 3′-UTR target sequence vectors and pre-miR negative control. Activities are shown relative to empty control vector (c) identically transfected and normalized as 3′-UTR target sequence vectors, Mann-Whitney U-test.

**Figure S1**
